# A panel of microRNA signature in serum for colorectal cancer diagnosis

**DOI:** 10.18632/oncotarget.15059

**Published:** 2017-02-03

**Authors:** Mingxia Zhu, Zebo Huang, Danxia Zhu, Xin Zhou, Xia Shan, Lian-wen Qi, Lirong Wu, Wenfang Cheng, Jun Zhu, Lan Zhang, Huo Zhang, Yan Chen, Wei Zhu, Tongshan Wang, Ping Liu

**Affiliations:** ^1^ Department of Oncology, The First Affiliated Hospital of Nanjing Medical University, Nanjing 210029, China; ^2^ Department of Radiation Oncology, The First Affiliated Hospital of Soochow University, Suzhou 215006, China; ^3^ Department of Oncology, The Third Affiliated Hospital of Soochow University, Changzhou 213003, China; ^4^ Department of Respiration, The Affiliated Jiangning Hospital of Nanjing Medical University, Nanjing 210009, China; ^5^ State Key Laboratory of Natural Medicines and Department of Pharmacognosy, China Pharmaceutical University, Nanjing, 210009, China; ^6^ Department of Radiation Oncology, Jiangsu Cancer Hospital, Nanjing 210009, China; ^7^ Department of Gastroenterology, The First Affiliated Hospital of Nanjing Medical University, Nanjing 210029, China; ^8^ Department of Emergency, The First Affiliated Hospital of Nanjing Medical University, Nanjing 210029, China; ^9^ Cancer Center of Nanjing Medical University, Nanjing 210029, China

**Keywords:** serum microRNA, colorectal cancer, diagnostic biomarker, qRT-PCR

## Abstract

Dysregulated expression of specific microRNAs (miRNAs) in serum has been recognised as promising diagnostic biomarkers for colorectal cancer (CRC). In the initial screening phase, a total of 32 differentially expressed miRNAs were selected by quantitative reverse transcription polymerase chain reaction (qRT-PCR) based Exiqon panel with 3 CRC pool samples and 1 normal control (NC) pool. Using qRT-PCR, selected serum miRNAs were further confirmed in training (30 CRC VS. 30 NCs) and testing stages (136 CRC VS. 90 NCs). We identified that serum levels of miR-19a-3p, miR-21-5p and miR-425-5p were significantly higher in patients with CRC than in NCs. The areas under the receiver operating characteristic (ROC) curve of the three-miRNA panel were 0.86, 0.74 and 0.87 for the training, testing and the external validation stages (30 CRC VS. 18 NCs), respectively. Significantly, elevated expression of the three miRNAs was also observed in CRC tissues (n = 24). Furthermore, the expression levels of the three miRNAs were significantly elevated in exosomes from CRC serum samples (n = 10). In conclusion, we identified a serum three-miRNA panel for the diagnosis of CRC.

## INTRODUCTION

Colorectal cancer (CRC) is the third most common cancer and the second leading cause of cancer-related death all around the world with an estimated 1.2 million new cases and a half million deaths each year [1]. The 5-year survival for patients with early-stage CRC is nearly 90%, so this disease could be potentially cured if early diagnosed [2]. Colonoscopy is widely used in clinical practice, which is regarded as the gold standard for detecting CRC. However, it has several limitations such as invasive nature, high cost and a bothering bowel preparation. Additionally, its success depends on the skill and experience of the operators. Thus, its widespread application for CRC in large-scale screening is hampered [3]. On the other hand, less-invasive diagnostic methods, such as fecal occult blood testing (FOBT) [4] and carcinoembryonic antigen (CEA) screening [5] in blood are of limited value owning to poor sensitivity and specificity [6, 7]. In view of this clinical challenge, novel, reliable and non-invasive biomarkers for early diagnosis of CRC are pressing needed.

MicroRNAs (miRNA), a class of small non-coding RNAs of 19-22 nucleotides in length, function as post-transcriptional regulators by directly cleaving target messenger RNA (mRNA) or translational repression [8]. Numerous studies have demonstrated that miRNAs can be stably detected in serum or plasma and have the potential to be new biomarkers for early diagnosis of various types of cancers [9–12]. Additionally, several studies have revealed that some miRNAs are able to discriminate CRC patients from healthy controls and serve as serum biomarkers for CRC screening with high accuracy [13, 14]. However, these results lack consistencies because of different research methods and tested populations between laboratories.

In the present study, we performed a four-phase study to screen miRNAs in CRC serum samples. In the screening phase, we systematically analyze the miRNA expression profile in 3 peripheral serum pools from 30 CRC cases and 1 pooled sample from 10 controls using miRCURY platform. Differentially expressed miRNAs were subjected to individual qRT–PCR confirmation in 30 CRC patients and 30 healthy controls in the training stage. Then, in the validation phase, the identified miRNAs were further detected in additional samples, including 136 CRC patients and 90 healthy subjects. Meanwhile, the diagnostic potential and effectiveness of these miRNAs for the detection of CRC was evaluated in an independent cohort of 30 CRC serum samples and 10 NCs. Furthermore, the expression profile of identified miRNAs was assessed in the CRC tissue. Serum exosomal miRNAs were further analyzed to explore the potential form of the identified miRNAs in serum of CRC patients.

## RESULTS

### Characteristics of study subjects

**Figure 1:**
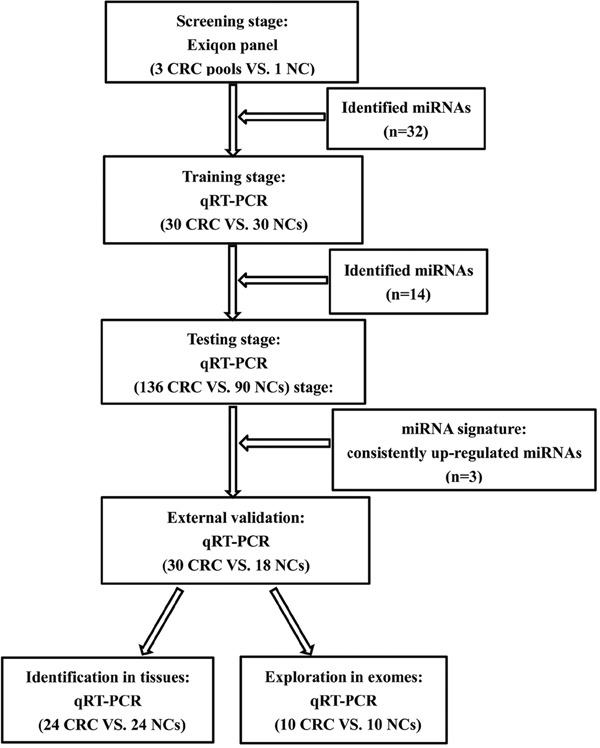
The flow chart of the experiment design. CRC: colorectal cancer; NC: normal control

A total of 334 serum samples from 196 patients with CRC and 138 healthy subjects were analyzed in a four-step approach that included: a screening phase, a training phase, a testing phase, and an external validation phase. The demographics and clinical features of the study subjects were shown in Table 1. Schematic representation of the experiment was illustrated in Figure 1. There were no significant differences between CRC patients and normal controls in the distribution of age or gender (*p* > 0.05).

**Table 1:** Clinical characteristics of 196 CRC patients and 138 normal controls

Variables	Training stage (n = 60)		Testing stage (n = 226)		External validation stage(n = 48)	
	Cases (%)	Controls(%)	Cases (%)	Controls(%)	Cases (%)	Controls(%)
**Number**	30	30	136	90	30	18
**Gender**						
Male	18(60)	18(60)	82(60.3)	46(51.1)	23(76.7)	10(55.6)
Female	12(40)	12(40)	54(39.7)	44(48.9)	7(23.3)	8(44.4)
**Age**						
<60	9(30)	22(73.3)	55(40.4)	48(53.3)	19(63.3)	11(61.1)
≥60	21(70)	8(26.7)	81(59.6)	42(46.7)	11(36.7)	7(38.9)
**Location**						
Colon	10(33.3)		61(44.9)		13(43.3)	
Rectum	20(66.7)		75(55.1)		17(56.7)	
**Differentiation grade**						
Middle-Low	28(93.3)		127(93.4)		29(96.7)	
High	2(6.7)		9(6.6)		1(3.3)	
**TNM stage**						
I	9(30)		26(19.1)		4(13.3)	
II	11(36.7)		60(44.1)		18(60)	
III	10(33.3)		50(36.8)		8(26.7)	
IV	0(0)		0(0)		0(0)	

### Profiling of serum miRNAs for CRC detection

We utilized the Exiqon miRCURY-Ready-to-Use-PCR-Human-panel-I + II-V1.M to screen the expression levels of 168 miRNAs in 3 serum pools from 30 CRC cases and 1 pooled sample from 10 controls. miRNAs that had a Ct < 37 and 5 Ct less than the negative control (No Template Control, NTC) and showed more than 1.5 fold altered expression between all 3 pooled CRC samples and the NC pool sample were selected as candidates. Among the 168 serum miRNAs detected, 32 miRNAs (29 up-regulated miRNAs and 3 down-regulated miRNAs; Supplementary Table 1) were found to be differentially expressed in CRC, which were further analysed by qRT-PCR in the subsequent validation step.

### Validation of miRNAs in serum by qRT-PCR

**Figure 2:**
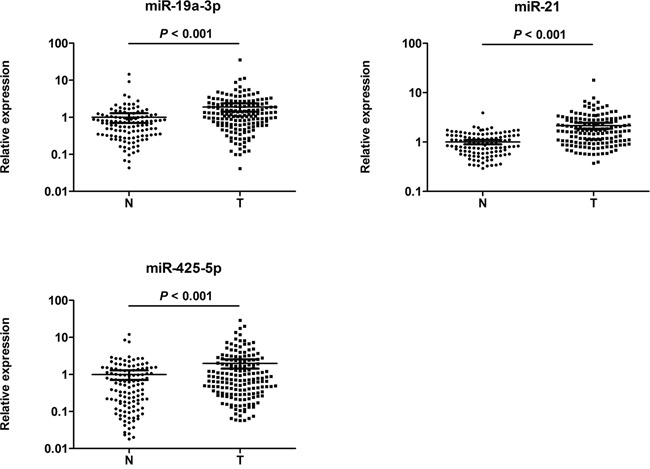
Expression levels of the three miRNAs in the serum of 166 CRC patients and 120 NCs (in the training and validation phases) A: miR-19a-3p; B: miR-21-5p; C: miR-425-5p; N: normal controls; T: tumor. Horizontal line: mean with 95% CI. The y axis represents relative expression of miRNAs normalized to cel-miR-39. *P*-values were calculated using the nonparametric Mann– Whitney U-test.

We next performed qRT-PCR assay to confirm the expression of 32 candidate miRNAs in 30 CRC patients and 30 NCs in the training stage. Fourteen miRNAs were found differentially expressed and selected for validation in a larger population consisting of 136 serum samples of CRC patients and 90 NCs in the testing phase (Supplementary Table 1). Consistent with the results from the training phase, 3 out of the 14 miRNAs (miR-19a-3p, miR-21-5p and miR-425-5p) showed significantly higher serum levels in CRC group than in control group. The differential expression of the three miRNAs in the 166 CRC samples compared to the 120 controls enrolled in the training and testing sets was shown in Figure 2.

### Diagnostic value of the candidate miRNAs

**Figure 3:**
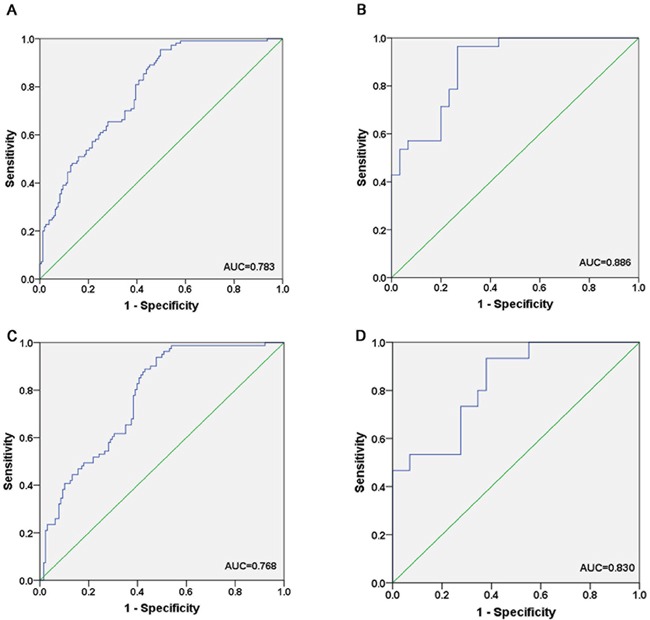
Receiver-operating characteristic (ROC) curves for the three-miRNA panel to discriminate CRC patients from NCs **A**. the combined two phases of training and validation phases (166 CRC VS. 120 NCs); **B**. training phase (30 CRC VS. 30 NCs); **C**. validation phase (136 CRC VS. 90 NCs); **D**. external validation (30 CRC VS. 18 NCs). AUC: area under the curve.

We generated ROC curves to evaluate the performance of the three miRNAs in discriminating the CRC patients from NCs. Logistic regression model for CRC prediction was applied on the data from the combination of training and validation phases. The AUC for serum miR-19a-3p, miR-21-5p and miR-425-5p was 0.685, 0.773 and 0.614, respectively when the training and validation samples were combined (Supplementary Figure 1). The combination of the three miRNAs presented further improvement in AUC of 0.783 (95% CI, 0.730 to 0.837) (Figure 3A) for patients with CRC. We also explored the diagnostic value of the three-miRNA panel in the two stages separately. For the training phase, the AUC of the three-miRNA panel was 0.886 (95% CI, 0.803 to 0.968) (Figure 3B). For the validation phase, the miRNA panel showed a AUC value of 0.768 (95% CI, 0.706 to 0.831) (Figure 3C).

To further validate the diagnostic performance of the three-miRNA panel, we measured the expression of miR-19a-3p, miR-21-5p and miR-425-5p by using qRT-PCR in an independent cohort of 30 CRC serum samples and 18 NCs. The results showed that all the three miRNAs were consistently overexpressed in the CRC group compared to NCs. Furthermore, the combination of these three miRNAs yielded an AUC of 0.830 (95% CI, 0.708 to 0.952) (Figure 3D), supporting the diagnostic value of this miRNA panel. We further evaluated the expression levels of miR-19a-3p, miR-21-5p and miR-425-5p with different TNM stages in the total training and validation phases. Unfortunately, none of the three miRNAs was significantly associated with clinical TNM stage (data not shown).

In addition, to evaluate whether these miRNAs were consistently differentially expressed in colon and rectal cancers, we conducted a subgroup analysis. As a result, miR-19a-3p, miR-21-5p and miR-425-5p were all up-regulated both in colon and rectal cancers. Moreover, miR-122-5p was found upregulated in colon cancers while miR-92a-3p upregulated in rectal cancers. The expression levels and diagnostic value of miR-122-5p, miR-19a-3p, miR-21-5p and miR-425-5p in colon cancers were shown in Supplementary Figure 2. The elevated expression and diagnostic performance of miR-19a-3p, miR-21-5p, miR-425-5p and miR-92a-3p in rectal cancers were shown in Supplementary Figure 3. miR-122-5p and miR-92a-3p might have the specificity in colon and rectal cancers, respectively.

### Expression of serum miRNAs in tissue samples

**Figure 4:**
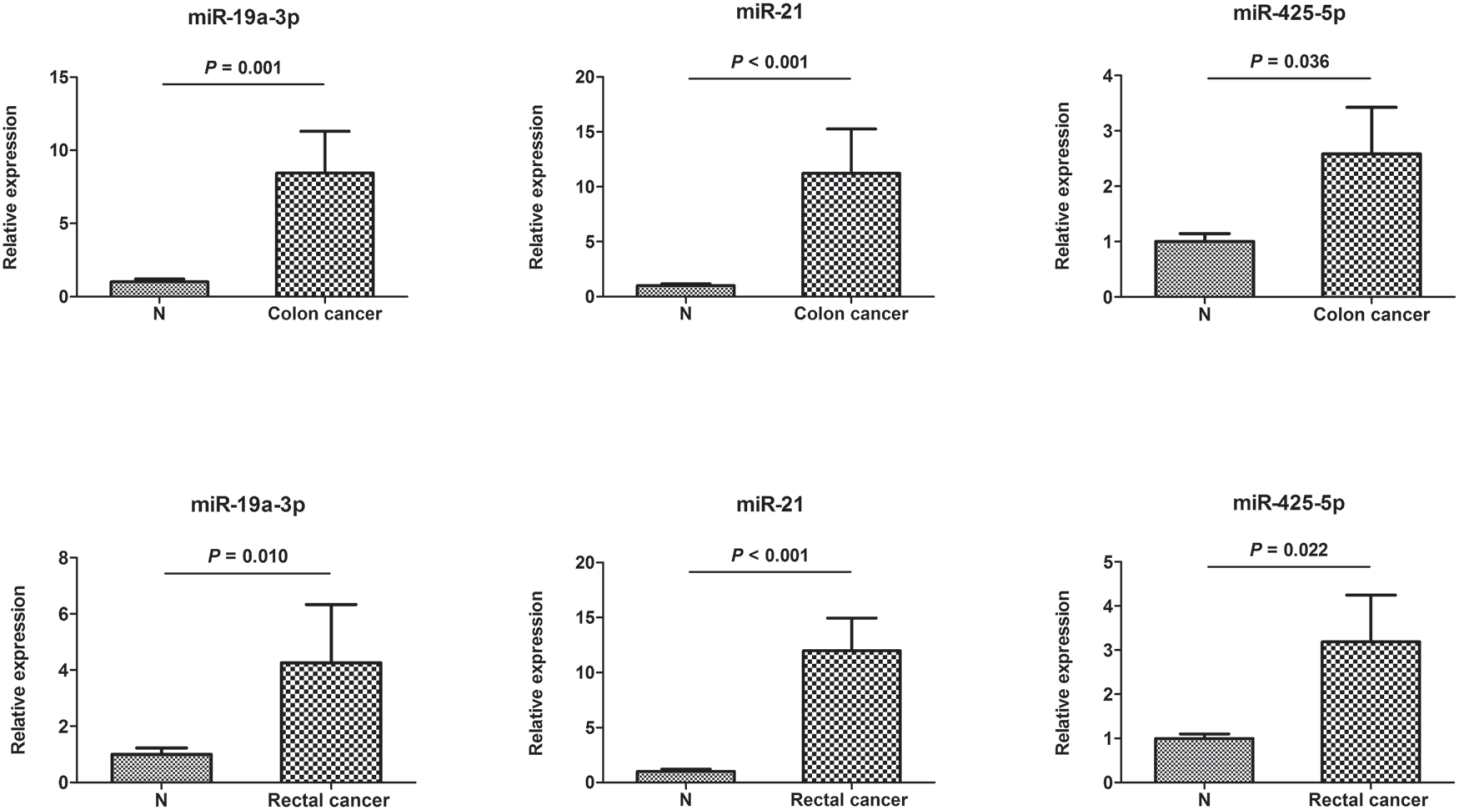
Expression of the three miRNAs in the tumor tissues of 12 colon and 12 rectal cancer patients N: adjacent nontumor tissues. Error bar: standard error. The y axis represents relative expression of miRNAs normalized to U6.

The three miRNAs identified in serum were further detectable using qRT–PCR in an additional of 24 pairs of FFPE tissue samples. Consistent with our serum results, the expression of miR-19a-3p, miR-21-5p, and miR-425-5p was found to be significantly higher in colon and rectal cancer tissues, respectively compared to those in normal tissues (Figure 4).

We also detected the expression levels of miR-122-5p and miR-92a-3p. The expression of the two miRNAs was significantly increased in colon and rectal cancer group compared with normal group, respectively (Supplementary Figure 4).

### Expression of the identified miRNAs in serum exosomes

**Figure 5:**
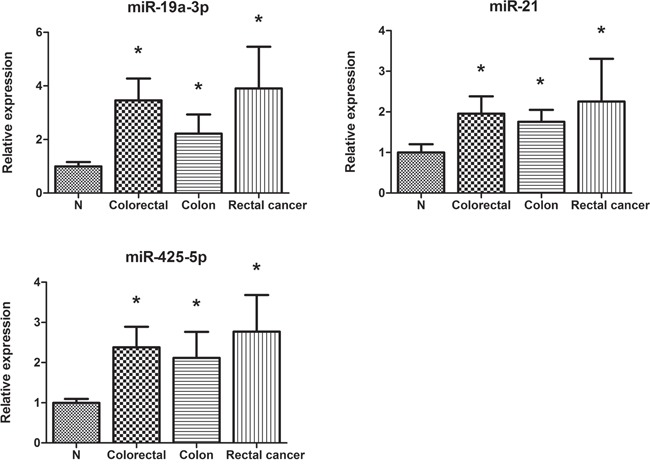
Expression of the three miRNAs in the serum exosomes of 10 CRC patients and 10 NCs Error bar: standard error.

The expression of exosomal miR-19a-3p, miR-21-5p, and miR-425-5p was assessed by qRT–PCR in 10 serum samples from CRC patients and 10 NCs to explore the potential form of the identified miRNAs in serum of CRC patients. Compared to NCs, all the three miRNAs were up-regulated in CRC serum exosomes. Additionally, the three miRNAs were consistently expressed in colon and rectal cancers (Figure 5).

The expression of miR-122-5p and miR-92a-3p were also found to be significantly higher in CRC serum exosomes compared to NCs. And the two miRNAs were also consistently expressed in colon and rectal cancers (Supplementary Figure 5).

### Comparison of miRNAs in peripheral and arterial serum

As blood flows from arterial to venous circulation, we assessed expression levels of the miRNAs in 5 arterial serum samples and compared those in the matched peripheral serum samples from the same individual. Among the three miRNAs, miR-21-5p showed higher expression levels in arterial plasma in more than a half of the subjects. However, due to the relatively small sample size, the results were not statistically significant (Supplementary Figure 6).

## DISCUSSION

In this study, we analyzed a cohort of 334 serum samples from 196 patients with CRC and 138 healthy subjects in a four-step approach, and found that the expression levels of miR-19a-3p, miR-21-5p and miR-425-5p could predict CRC. A few recent studies have revealed the diagnostic role of circulating miRNAs in CRC [13, 14, 18]. Unfortunately, to date, independent studies have failed to develop clinically actionable miRNA biomarkers for a variety of reasons including population and sample diversity, especially the discovery and validation methodologies used in these reports. In the current study, we utilized Exiqon miRNA qPCR panels which appeared to show better sensitivity and linearity with measurements of miRNAs in relatively low abundance than TaqMan platform [19]. This platform was used in the screening phase to analyze differential expression profiling of serum miRNAs in 3 CRC and 1 NC pooled samples. The screening stage was followed by the training and testing phases of RT–qPCR validation in our study. We found that up-regulated levels of miR-19a-3p, miR-21-5p and miR-425-5p in serum could accurately discriminate patients with CRC from healthy controls. To ensure the reliability and reproducibility of the diagnostic value of the three -miRNA signature, we intensively validated our findings in the external validation stage. Notably, the expression of miR-19a-3p, miR-21-5p and miR-425-5p in CRC tissue samples was consistent with our serum results, which suggested the important roles for these miRNAs in tumorigenesis and progression of the disease.

Serum miR-19a-3p has been explored as a potentially noninvasive biomarker for colorectal cancer in two additional studies [14, 20]. As a member of the miR-17-92 cluster, miR-19a-3p could promote the proliferation and metastasis of colon cancer cells [21]. Another study reported that miR-19a-3p could enhance invasion and metastasis by targeting Transglutaminase-2 in CRC cells [22]. Serum miR-19a-3p could predict resistance to FOLFOX chemotherapy in advanced colorectal cancer cases [23]. miR-21-5p has been reported as a reliable diagnostic biomarker for colorectal cancer in several additional studies that is in accordance with our finding [13, 18, 24, 25]. Recent findings indicate that miR-21-5p might function as oncogene in various cancers, including CRC. miR-21-5p could regulate several different target genes and pathways involving tumor proliferation, invasion and metastasis and play an important role in 5-FU resistance and affect the radiosensitivity of CRC cells [26–28]. Especially, serum miR-21-5p level was found to be a robust independent predictor of the presence of CRC [29]. However, there was no significant association between serum miR-21-5p levels and clinical stage in patients with CRC in our study. miR-425-5p has been reported to be implicated in tumorigenesis in many cancer types [30–32]. However, the mechanism of miR-425-5p in CRC is still unclear. Zhang et al. showed that miR-425-5p could regulate chemoresistance in CRC cells via regulating Programmed Cell Death 10 [33]. In addition, miR-425-5p has been reported to promote tumorigenicity and aggressiveness in breast cancer and gastric cancer [31, 34]. miR-425-5p was discovered for the first time to be valuable biomarker of CRC in our study. The function of miR-425-5p in CRC is needed to be further investigated. We also found that serum miR-122-5p was up-regulated in colon cancer. miR-122-5p, a liver-specific miRNA that is abundant in the liver and plays an important role in regulating hepatocyte development and differentiation [35]. miR-122-5p has also been reported to regulate tumorigenesis in hepatocellular carcinoma and have tumor-suppressive function in breast cancer [36, 37]. Additionally, miR-92a-3p was upregulated in rectal cancer not colon cancer serum. miR-92a-3p was evaluated as a diagnostic biomarker in CRC in previous study [14, 38, 39]. As a member of large cluster miR-17-92, miR-92a-3p has an important role as an oncogene in some cancers [40, 41]. Upregulation of the miR-92a-3p has been shown to disrupt the functions of several important factors of growth and division in colorectal cells [42]. Schee K et al. suggested that miR-92a-3p was involved in the metastasis of CRC by targeting the CDH1 gene [43]. miR-122-5p and miR-92a-3p may be as potentially biomarker for discriminating colon cancer from rectal cancer. Interestingly, the five miRNAs expression were found to be consistently higher in colon and rectal cancer tissues, while the analysis of miRNA expression by The Cancer Genome Atlas (TCGA) project identified no clear distinctions between rectal cancers and colon cancers tissues [44], which suggested that there might be some difference of the miRNA expression profile between serum and tissue samples in CRC. Of course, further research of these miRNAs in CRC formation and development is needed.

Certainly, these circulating miRNAs identified in our study were also associated with some other cancers. High serum miR-19a-3p expression correlates with worse prognosis of patients with non-small cell lung cancer [45]. miR-21-5p acts as a broad-spectrum biomarker for many other solid cancers, including gastric, pancreatic, breast, and prostate cancers [11, 46–48]. One advantage characteristic for the serum miR-21-5p level is its relatively higher sensitivity and specificity in diagnosis of CRC than both NCs and other cancer patients [49, 50]. miR-425-5p has been identified as a potential biomarker in renal cell carcinoma, lung squamous cell carcinoma, breast cancer and bladder cancer [30, 51, 52]. An up-regulation of circulating miR-425-5p has been observed in head and neck cancer patients after radiotherapy in the blood plasma compared with primary HNSCC (head and neck squamous cell carcinoma) cells [53]. This is a common concern with many other miRNA biomarkers, because of their simultaneous dysregulation in multiple cancers. Belonging to circulatory system, arterial blood is more difficult to obtain. We assessed the expression levels of the three miRNAs in arterial serum of only 5 patients. Unfortunately, no result achieved statistical significance due to the small sample size. Apparently, more efforts were needed to investigate the specificity of circulating miRNAs.

Given the fact that the majority of circulating miRNAs come from tumor tissue [54], we evaluated the expression of the three identified serum miRNAs in CRC tissues. It turned out that miR-19a-3p, miR-21-5p and 425-5p were found to be highly upregulated in CRC tissues compared to those in normal tissues. The findings may at least in part verify the theory. Exosomes are small membrane vesicles of approximately 30–140 nm that embed protein, lipids, mRNAs, and miRNAs, depending on the origin of the secreting cells [55]. A recent study discovered that miRNA expression in serum and saliva was predominantly derived from exosomes [56]. Moreover, recent studies demonstrated that exosomal miRNAs in serum may be promising diagnostic biomarkers for the detection of CRC [57]. We further explored serum exosomal miRNAs to identify the potential form of the three miRNAs in serum. miR-19a-3p, miR-21-5p, and miR-425-5p were up-regulated in CRC serum exosomes. miR-122-5p and miR-92a-3p were also found to be significantly higher in CRC exosomes compared with NCs. Other studies have showed that the majority of miRNA signal in serum is primarily contributed by free-floating RNAs. More studies are needed to resolve this issue.

In conclusion, a three-miRNA panel in the serum of CRC patients which could serve as a non-invasive biomarker in the detection of CRC was identified and validated. Our serum based miRNA biomarkers may be clinically applicable for noninvasive screening of patients with CRC.

## MATERIALS AND METHODS

### Study design, patients and controls

A total of 196 histopathologically conformed CRC patients and 138 normal controls (NCs) were enrolled between 2012 and 2014 at the First Affiliated Hospital of Nanjing Medical University (training and testing stage) and the Third Affiliated Hospital of Soochow University (external validation stage). People who showed no evidence of disease (including cancer, precancerous lesion and chronic diseases) were selected as normal controls. An additional of twenty-four paired formalin fixed paraffin embedded (FFPE) sections of CRC and matched adjacent gastric mucosa tissues were obtained. All the procedures were approved by Institutional Review Boards of the First Affiliated Hospital of Nanjing Medical University. Written informed consent was taken from each participant.

In this study, we carried out a 4-stage study for CRC. In the initial screening stage (Figure 1), thirty peripheral serum samples from CRC patients and 10 from NCs were randomly chosen and pooled as 3 CRC samples and 1 NC sample (10 samples were pooled as 1 pool sample, colon-to-rectal ratio 33 to 23) for miRNA microarrays. Approximately 20–25 ng RNA isolated from each pool of serum samples was reverse transcribed to cDNA by using the miRCURY Locked Nucleic Acid (LNA™) Universal Reverse Transcription (RT) microRNA PCR, Polyadenylation and cDNA synthesis kit (Exiqon miRNA qPCR panel, Vedbaek, Denmark) following the manufacturer’s protocol. Microarrays were scanned on 7900HT real-time PCR system (Applied Biosystems, Foster City, CA, USA) with Exiqon miRCURY-Ready-to-Use PCR-Human-panel-I + II-V1.M (Exiqon miRNA qPCR panel, Vedbaek, Denmark), which could detect 168 miRNAs in plasma/serum to identify differently expressed miRNAs. Melting curve analyses were performed at the end of the PCR cycles. Detectable miRNAs were those with a Ct < 37 and 5 Ct less than the negative control (No Template Control, NTC). An RNA spike-in (UniSp6) and a DNA spike-in (Sp3) were used as technical controls to evaluate if the technical performance of all samples is similar. The Ct values were normalized based on the average of the normalizer assays in the panel and this included miR-423-5p and miR-93-5p. The formula used to calculate the normalized Ct values is: normalized Ct (Δ Ct) = average Ct (assay) – average Ct (normalizer assays). The relative expression levels of miRNAs between CRC patients and NCs were calculated using 2^-ΔΔCt^ method.

In the training stage, the differentially expressed miRNAs discovered via screening phase was confirmed using qRT-PCR in 30 CRC samples and 30 NCs. Then, the miRNAs identified by the training stage were further evaluated by qRT-PCR in the testing phase in serum samples including 136 CRC patients and 90 NCs. For the external validation set, we subjected 30 cases and 18 controls to evaluate the diagnostic value of the three-miRNA signature in CRC. The selected miRNAs were further verified in 12 pairs of formalin-fixed paraffin-embedded (FFPE) colon cancer tissue specimens and 12 pairs of rectal ones and adjacent nontumor tissues from surgery patients. In addition, exosomal miRNAs from 10 patients and NCs were analyzed to investigate the potential form of the miRNAs in the peripheral serum.

Whole blood samples (5ml) were collected from each patient and normal controls with overnight fasting. In CRC patients, blood samples were collected within one week before initial treatment. All samples were processed within 1 hour and stored in 4°C, then subjected to centrifuge at 1,500 r.p.m. for 10 min within 12 h after collection. Then, cell-free serum was further resolved by centrifugation at 1,2000 r.p.m. for 2 min to guarantee complete removal of cell debris. The serum sample was stored in an RNase-free eppendorf tube at -80°C until use.

### RNA extraction

RNA was isolated from 200μl serum using mirVana Paris Kit (Ambion, Austin, TX, USA) according to the manufacturer’s protocol. To allow for normalization of sample-to-sample variation in RNA extraction procedures, synthetic C. elegans miRNA cel-miR-39 (5 nM/L, 5μl RiboBio, Guangzhou, China) was spiked into each denatured sample after combining the serum sample with denaturing solution (Ambion, Austin, TX, USA). Total RNA was extracted from FFPE specimens using the High Pure FFPE RNA Micro Kit (Ambion, Austin, TX, USA). RNA was eluted with 100μl of RNase-free water and stored at − 80°C for further use. The ultraviolet spectrophotometer was used to evaluate the concentration and purity of the total RNA. The concentration of serum RNA ranged from 15.73 ng/μl to 48.62 ng/μl.

### Isolation of exosomes

ExoQuick Exosome Precipitation Solution (System Biosciences, Mountain View, Calif)was used to isolate exosomes from serum according to the manufacturer’s protocol. Briefly, 200μl serum was mixed with 100μl ExoQuick exosome precipitation solution and then kept at 4°C for 30 min, followed by centrifugation at 13,000 rpm for 2 min. After the supernatants were removed, the exosome pellets were retained for further RNA extraction.

### Quantitative reverse transcription polymerase chain reaction (qRT-PCR)

The amplification of miRNA was performed using the specific primers of reverse transcription (RT) and polymerase chain reaction (PCR) from Bulge-Loop™ miRNA qRT-PCR Primer Set (RiboBio, Guangzhou, China) as previously described [15, 16]. The quantification of PCR product was evaluated by the level of fluorescence in emitted by SYBR Green (SYBR^®^ Premix Ex Taq™ II, TaKaRa). RT and PCR were performed as previously described [15]. RT reactions were carried out at 42°C for 60 min followed by 70°C for 10 min. The qRT-PCR was run on a LightCycler® 480 Real-Time PCR System (Roche Diagnostics, Mannheim, Germany) in 384-well plates at 95°C for 20 sec, followed by 40 cycles of 95°C for 10 sec, 60°C for 20 sec and then 70°C for 10 sec. The melting analysis was added finally to evaluate the specificity of PCR products. The expression of miRNAs in tissue specimens, serum samples and exosomes were calculated using the comparative 2^-ΔΔCt^ method relative to *RNU6B* (*U6*) and cel-miR-39 [17].

### Statistical analysis

Differential miRNAs expression between CRC patients and NCs was analyzed using Mann-Whitney test. The association between miRNAs and the clinical characteristics was estimated by the one-way ANOVA or χ2 test. Receiver operating characteristic (ROC) curves and the area under the ROC curve (AUC) were used to evaluate the diagnostic value of the candidate miRNAs for CRC. Logistic regression model for CRC prediction was applied on the data from the training and validation phases. SPSS (version 15.0, SPSS Inc., Chicago, IL, USA) software was used to perform all statistical analysis. Differences were considered significant when *p* < 0.05.

## SUPPLEMENTARY FIGURES AND TABLE


