# Supplementary material for: A panel of microRNA signature in serum for colorectal cancer diagnosis
Source: Oncotarget. 2017 Feb 3;8(10):17081–91. doi: 10.18632/oncotarget.15059 (PMC5370024; doi:10.18632/oncotarget.15059)
Supplement: Supplementary file 1 [file oncotarget-08-17081-s001.pdf]

A panel of microRNA signature in serum for colorectal cancer diagnosis

SUPPLEMENTARY FIGURES AND TABLE

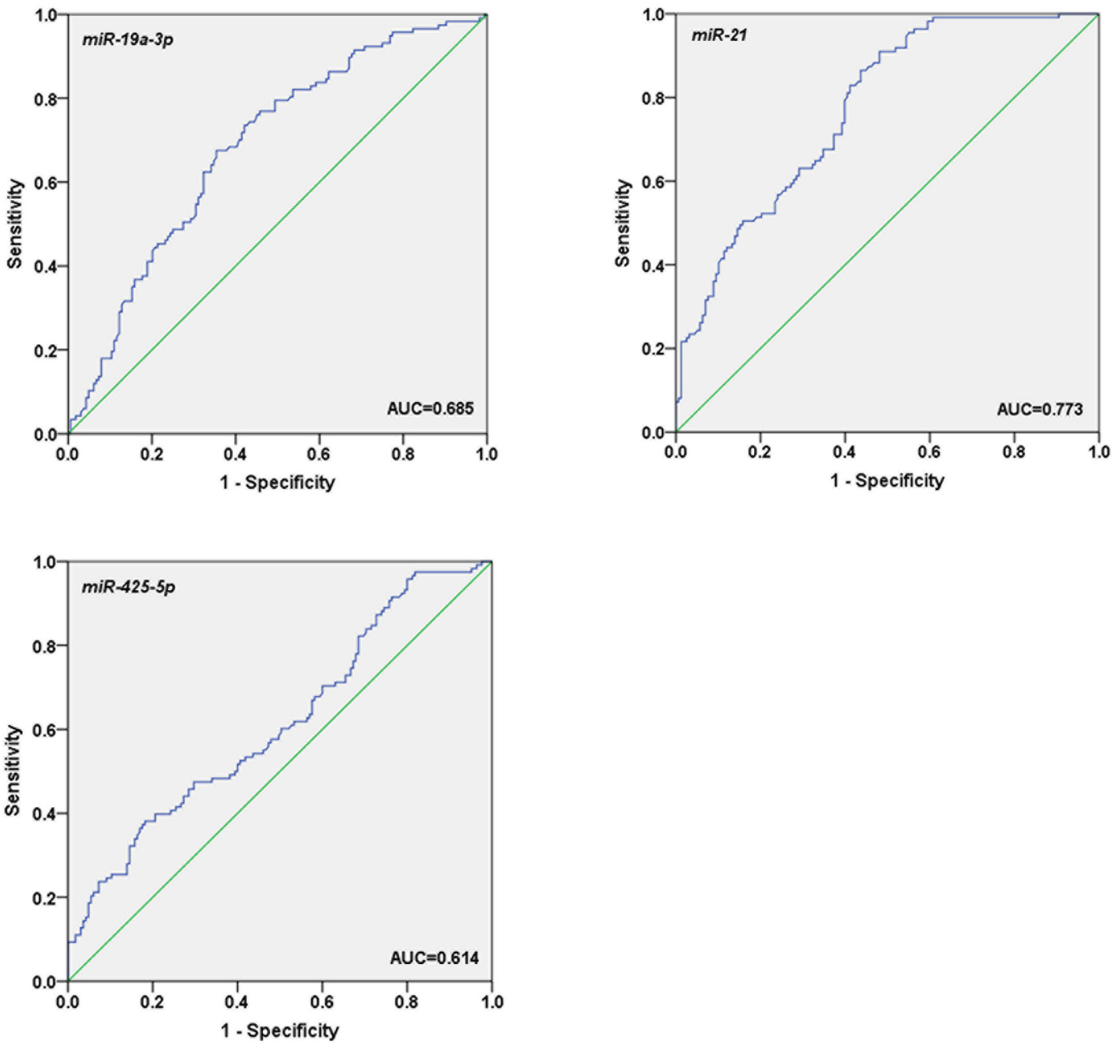

Supplementary Figure 1: ROC curve analyses of each miRNA to discriminate CRC patients from NCs in the combined two phases.

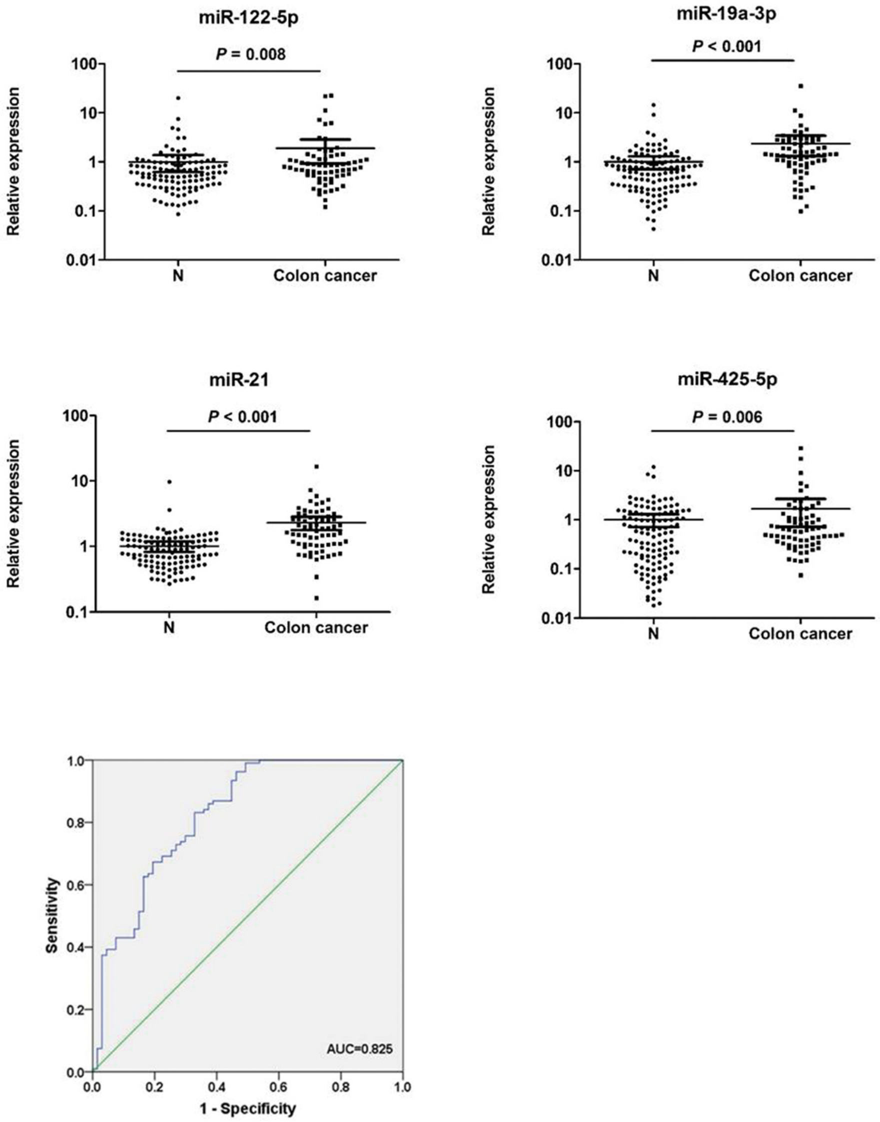

Supplementary Figure 2: Expression levels of miR-122-5p, miR-19a-3p, miR-21-5p and miR-425-5p and ROC curve analyses of the four miRNAs in colon cancers. (in the training and validation phases).

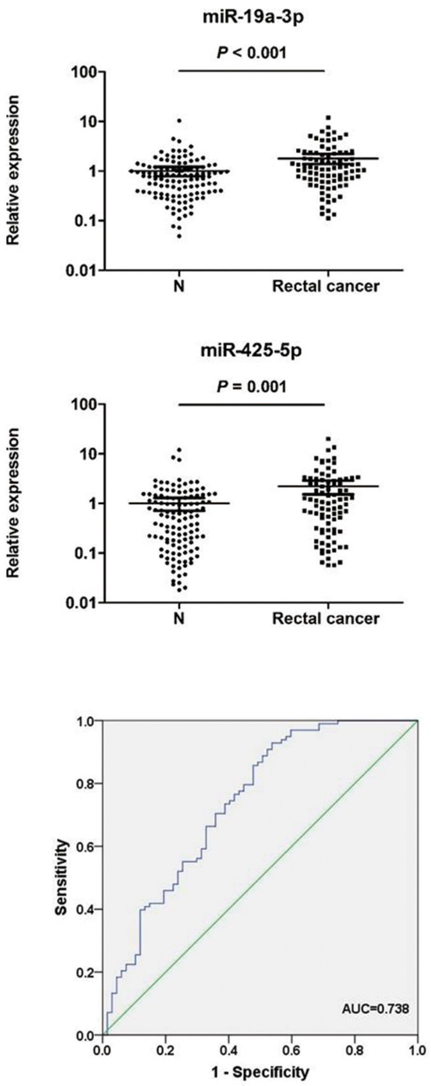

Supplementary Figure 3: Expression levels of miR-19a-3p, miR-21-5p, miR-425-5p and miR-92a-3p and ROC curve analyses of the four miRNAs in rectal cancers. (in the training and validation phases).

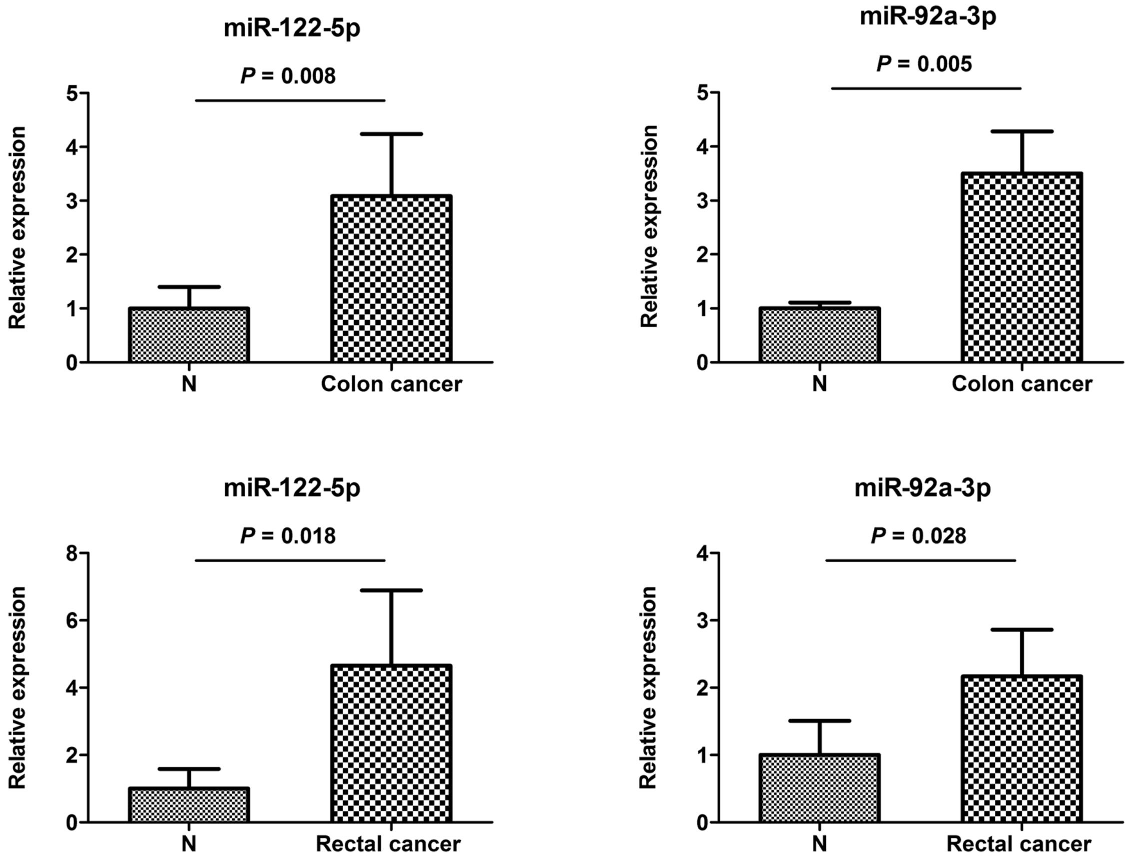

Supplementary Figure 4: Expression of miR-122-5p and miR-92a-3p in the tumor tissues of 12 colon and 12 rectal cancer patients. N: adjacent nontumor tissues. Error bar: standard error.

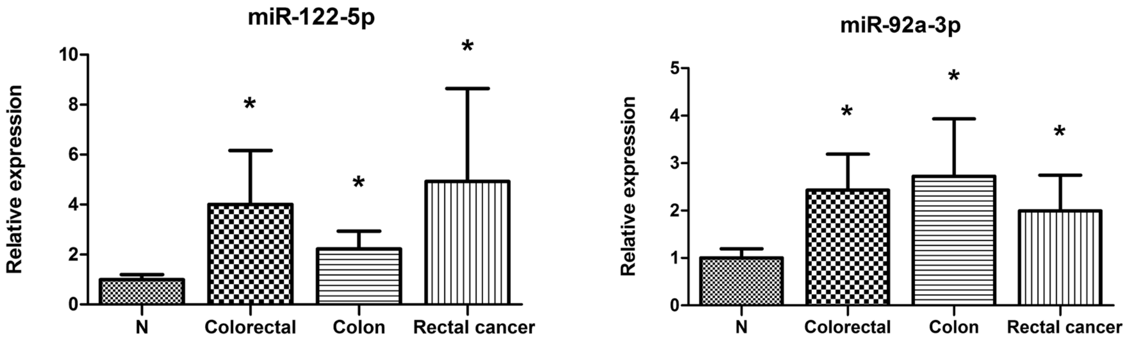

Supplementary Figure 5: Expression of miR-122-5p and miR-92a-3p in serum exosomes of 10 CRC patients and 10 NCs. N: normal controls; T: tumor. Error bar: standard error.

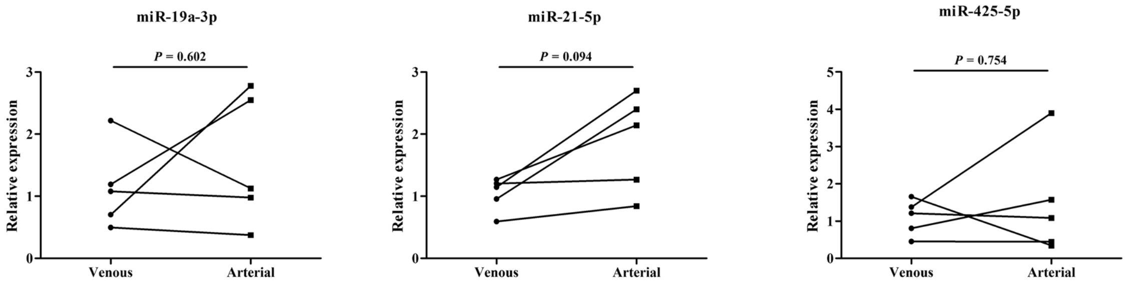

Supplementary Figure 6: Comparison of the three miRNAs in 6 arterial serum samples and matched peripheral serum samples.

Supplementary Table 1: Differently expressed miRNAs in the screening phase

MiRNA	Fold change			Mean fold
	Pool 1	Pool 2	Pool 3	
miR-10b-5p	31.07	2.40	5.65	13.04
**miR-122-5p**	10.56	15.67	5.59	10.61
**miR-130a-3p**	8.43	5.01	3.65	5.70
miR-136-5p	6.00	4.02	4.70	4.91
**miR-140-5p**	3.86	8.87	6.27	6.33
miR-141-3p	19.94	3.13	8.27	10.45
miR-144-5p	10.57	7.26	2.04	6.62
**miR-18a-5p**	4.59	2.03	2.74	3.12
**miR-19a-3p**	5.12	2.87	2.57	3.52
**miR-20a-5p**	7.66	3.80	3.08	4.85
**miR-210-3p**	2.77	4.35	3.07	3.40
**miR-21-5p**	6.92	4.17	4.59	5.23
**miR-29b-2-5p**	7.39	4.65	3.97	5.34
**miR-425-5p**	4.58	3.95	1.97	3.50
**miR-92a-3p**	3.59	3.05	1.83	2.82
miR-144-3p	9.19	5.30	3.61	6.03
miR-185-5p	5.13	3.18	3.06	3.79
miR-146b-5p	4.11	6.97	5.44	5.51
miR-155-5p	4.49	19.50	6.31	10.10
miR-18b-5p	2.41	4.73	3.51	3.55
miR-192-5p	18.04	11.30	5.14	11.49
miR-194-5p	12.46	13.12	4.52	10.03
miR-215	12.58	10.21	7.39	10.06
**miR-223-5p**	-2.01	-2.28	-1.52	-1.94
**miR-29a-3p**	4.27	3.31	3.77	3.78
miR-296-5p	44.68	7.12	2.47	18.09
miR-34a	6.65	4.45	3.00	4.70
miR-361-3p	4.17	4.70	2.69	3.85
**miR-382-5p**	-1.89	-2.06	-1.56	-1.84
miR-500a-5p	-12.36	-18.44	-21.56	-17.45
miR-95	18.20	11.10	16.25	15.18
miR-99a-5p	9.23	11.70	3.90	8.28

Note: miRNAs in bold and underline were identified after the training phase.
